# Early access disparities in innovative therapies across the US, EU, China, and Japan

**DOI:** 10.3389/fmed.2025.1642882

**Published:** 2025-10-23

**Authors:** Sanae Akodad, Michel Goldman, Hilde Stevens

**Affiliations:** Institute for Interdisciplinary Innovation in Healthcare (I3h), Faculté de Médecine, Solvay Brussels School of Economics and Management, Université Libre de Bruxelles (ULB), Brussels, Belgium

**Keywords:** early access, unmet medical need, regulatory disparities, health technology assessment (HTA), global health equity

## Abstract

Early access to innovative therapies offers a critical lifeline for patients with severe or life-threatening conditions, yet substantial disparities persist across the United States (US), the European Union (EU), China (CH), and Japan (JP). These disparities arise from diverging definitions of unmet medical need (UMN), heterogeneous timelines for regulatory review, and uneven implementation of Early and Expanded Access mechanisms. This policy review provides a comparative analysis of regional frameworks, emphasizing how clinical trial geography, eligibility rules, and access mechanisms interact to shape pre-approval opportunities for patients. While regulatory innovations such as the FDA's Breakthrough Therapy designation, the EMA's PRIME scheme, the PMDA's Sakigake program, and recent reforms in China have accelerated access in selected contexts, cross-border misalignments and fragmented health technology assessment (HTA) processes continue to generate inequities. The EU's new HTA Regulation (2021/2282) represents a step forward by embedding joint clinical assessment and real-world evidence, but persistent structural inconsistencies hinder timely and equitable uptake. This article argues for a paradigm shift from static approval models to adaptive licensing approaches, including live licenses and regulatory sandboxes. It calls for internationally coordinated benefit–risk governance that integrates early access planning into drug development from Phase II onward. By reframing uncertainty as a shared responsibility among regulators, developers, payers, clinicians, and patients, early access governance can be redesigned to promote equity, transparency, and responsiveness particularly for rare and high-burden diseases.

## 1 Introduction

Patients facing an unmet medical need (UMN), defined variously across regulatory agencies, are often desperate to gain early access to innovative therapies that could alleviate their suffering.

While the FDA defines UMN as a condition where no satisfactory treatment exists or where existing treatments fail to produce adequate outcomes, the European Medicines Agency (EMA) emphasizes severity, rarity, and the absence of alternatives (1, 2). Meanwhile, Japan's Pharmaceuticals and Medical Devices Agency (PMDA) considers factors like disease progression and the availability of local treatment options in defining urgency (3). In China, the National Medical Products Administration (NMPA) has progressively aligned its criteria with international standards through a series of reforms since 2017, including the Opinions on Deepening the Reform of the Review and Approval System and the 2019 Drug Administration Law (4). These reforms introduced expedited pathways such as conditional approvals for drugs addressing serious or life-threatening diseases with no effective treatment, thereby incorporating the concept of UMN into Chinese regulatory practice (4–6).

In this article, *innovation* is considered only in relation to UMN. While a therapy may be innovative without responding to an UMN, and conversely, a therapy may meet an UMN without being formally classified as innovative, our focus is restricted to innovative products developed to address an UMN. This scope reflects the subset of therapies for which early access pathways are most consequential for patients and systems (7, 8). Such innovative therapies, aimed at meeting an UMN, are usually scientifically and technically complex (often biological) products, targeting specific—often small—patient populations, which has repercussions on the uncertainty of, and time needed to collect sufficient CT data, and consequently, affects the time required for companies to reach a (conditional) market authorization (MA) (9). The different regulatory framework characteristics, conditions and timelines are carefully assessed by CT sponsors and are decisive for the geographic location of the first CT set-up. This decision, and the existence of country-specific special Early Access Programs (EAPs) and Expanded Access (EA) mechanisms for patients not eligible to participate in a CT, are crucial for the patients' disease journey (10). It creates inequalities between patients and patient populations across different regions of the world. It has been recently highlighted that 52% of delays in patient access across the EU are attributable to the absence or lateness of local CT activity, suggesting that early decisions on trial geography have downstream consequences on access equity (11). These inequalities are no longer anecdotal, the EFPIA Patients W.A.I.T. indicator shows that between 2018 and 2022, many Central and Eastern European countries had markedly lower availability of EMA-authorized medicines, often with delays exceeding 500 days compared with Western Europe (12). For clarity, the main regulatory definitions of UMN, innovation, and early access mechanisms are summarized in Table 1.

**Table 1:** Regulatory definitions of UMN, innovation, and early access mechanisms.

**Agency**	**Definition of UMN**	**Definition of innovation**	**Early access mechanisms**
FDA (US)	No satisfactory alternatives or inadequate outcomes with existing therapies.	Significant improvement over available therapies (criterion for expedited programs).	Expanded Access (individual, intermediate, emergency); Accelerated Approval; Breakthrough Therapy; Fast Track; Priority Review.
EMA (EU)	Serious condition, rarity, and lack of satisfactory alternatives.	Major therapeutic advantage over existing options.	Compassionate Use Programs (CUPs); Named Patient Programs (NPPs); Conditional Marketing Authorization; Accelerated Assessment; PRIME.
PMDA (Japan)	Urgency based on disease progression and local treatment availability.	Therapies showing clear clinical benefit beyond available options.	Expanded Access Clinical Trials (EACTs); Priority Review; Sakigake Designation.
NMPA (China)	Severe or rare diseases lacking effective therapies (2017–2019 reforms).	Novel therapies with improved efficacy or safety over existing standards.	Conditional Approval; Priority Review; Hainan Boao Lecheng Pilot Zone (special access with RWD linkage).

The patient's access to innovative therapies is affected not only by CT sponsor's decisions, and the existence of special EAPs, but also by region- and country-specific delays further downstream the drug development lifecycle, namely differences in country-specific regulatory frameworks on pricing and reimbursement. Both regulatory MA as well as the country-specific pricing and reimbursement process depend on the benefit-risk assessment of the innovative therapy. The significance of CT data, and -where conditional approvals were agreed- the follow-up data is rigorously assessed by the regulatory authorities.

**Figure 1:**
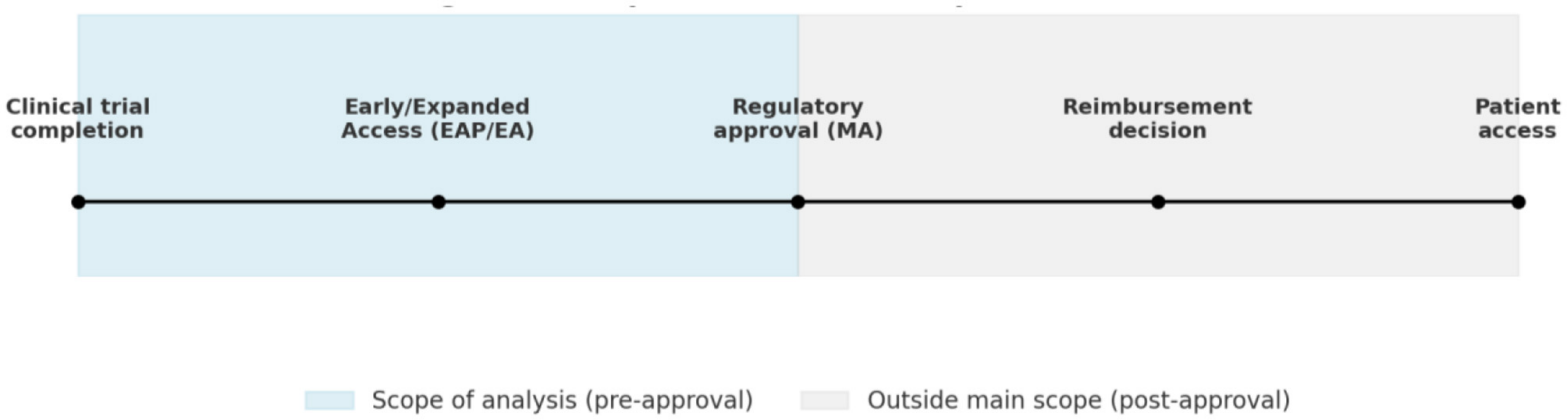
Temporal dimensions of patient access.

In comparative analyses of early access, the notion of *access time* requires precise delineation. For the purposes of this article, we distinguish three sequential dimensions: (i) the interval between clinical trial completion and marketing authorization (MA) (time-to-approval), (ii) the possibility of obtaining treatment through Early or Expanded Access (EAP/EA) mechanisms before formal approval (time-to-EAP/EA), and (iii) the period between regulatory approval and effective patient use following pricing and reimbursement decisions (time-to-reimbursement). Our analysis concentrates on the first two dimensions, which directly determine access opportunities before marketing authorization (Figure 1). Delays in reimbursement remain an equally important barrier, particularly in the EU and China where health technology assessment (HTA) and pricing negotiations often extend the interval before effective uptake, but these fall beyond the scope of our present analysis (12, 13).

By clarifying access times in this way, we align with previous comparative frameworks while situating our analysis at the pre-approval stage, where inequalities are most visible and where regulatory design exerts the greatest leverage (7, 8).

This article investigates pre-approval disparities in patient access to innovative therapies across four regulatory regions: the United States (US), the European Union (EU), China (CH), and Japan (JP). We examine how patient eligibility for clinical trials and the availability of EAP/EA mechanism interact with regulatory timelines to shape access opportunities before marketing authorization. These structural variations generate inequities in the timing of access to potentially life-altering therapies, with tangible consequences for patients living with severe or rare diseases.

## 2 Scope and analytical framework

This study applies a comparative policy analysis to examine disparities in early access to innovative medicines prior to marketing authorization. The focus is on four major regulatory regions—the United States (US), the European Union (EU), Japan (JP), and China (CH)—chosen for their global influence and contrasting health system architectures.

### 2.1 Sources of evidence

The analysis draws on regulatory documents (FDA, EMA, PMDA, NMPA), official policy reports and peer-reviewed literature published between 2018 and 2025. Documents were selected for their direct relevance to early access mechanisms and for their potential to allow cross-regional comparison.

### 2.2 Analytical dimensions

The comparison was structured around four interrelated domains. First, we considered the geography of clinical trials and its impact on local patient inclusion. Second, we examined the regulatory pathways for accelerated or conditional approval, as well as the design of early or expanded access programs. Third, we assessed how health system financing structures shape the feasibility of granting pre-approval access. Finally, we analyzed the mechanisms used to manage evidentiary uncertainty, with particular attention to the development of Managed Entry Agreements.

### 2.3 Comparative approach

By examining these domains across the four jurisdictions, the study identifies how regulatory design and health system structure interact to determine the timeliness and equity of patient access. Emphasis is placed on pre-approval mechanisms, since this is the stage at which disparities are most visible and where policy choices exert the greatest leverage.

### 2.4 Limitations

This analysis is based on purposive selection of regulatory and policy sources, rather than a systematic review of all available literature. As such, it privileges policy relevance and recency over exhaustive coverage. Patient-level quantitative data, particularly denominators of eligible populations in early access programs, remain limited in many jurisdictions, which constrains cross-regional comparability. Finally, post-approval outcomes such as reimbursement delays and long-term cost-effectiveness are not comprehensively assessed here, except where they directly influence pre-approval access.

## 3 Comparative analysis

### 3.1 Geographical disparities in clinical trials

CTs are the primary means through which patients can access new, potentially life-saving treatments long before they reach the market. Different regulatory agencies in the US (FDA), the EU (EMA), CH (NMPA) and JP (PMDA) have installed various accelerated pathways to speed up the review process of innovative therapies targeting UMN. However, the global distribution of CTs is not uniform. Certain regions host more trials, and pharmaceutical companies often initiate CTs in regions with faster regulatory review processes, leading to geographical disparities in access to cutting-edge treatments.

Although most pivotal trials are designed as multicentric, their initiation is rarely synchronous across all sites. Early-opening sites, typically concentrated in regions with streamlined regulatory and ethical approvals, provide local patients with months or even years of earlier access compared to countries activated later (7). Moreover, patients in countries without trial participation are effectively excluded from pre-approval access, unless alternative mechanisms such as EAPs are in place. Recent analyses indicate that 52% of delays in patient access in the EU can be attributed to the absence or lateness of local CT activity, underscoring the structural importance of geography (11). In CH, large-scale investment and accelerated site initiation have rapidly expanded local patient inclusion, while in JP the reliance on EACTs has provided limited but structured access for otherwise ineligible populations (3, 5, 6). These dynamics illustrate that CT geography is not neutral: the sequencing and density of trial sites directly shape who benefits earliest from innovative therapies.

The US no longer leads in CT activity, although it still represented around ≈23% of global commercial clinical drug trials in 2023 (11). In contrast, EU's share declined from 22% in 2013 to 12% in 2023, highlighting a significant reduction in its global CT footprint (11). This decline is attributed to lengthy initiation times and complex regulatory hurdles, which discourage rapid trial start-ups and exacerbate disparities between CT activities in the US and EU. Meanwhile, the Asia-Pacific (APAC) region has seen notable growth. JP, in particular, has strengthened its position as a preferred destination for high-quality trials, representing approximately 4.7% of global CT activity in 2022 (11). With well-established regulatory frameworks and initiatives, JP continues to attract innovative drug development, though it faces increasing competition from other APAC countries. Among these, CH has emerged as the dominant global player, accounting for 29% of all clinical drug trials in 2023, more than doubling its share since 2018 (5, 6). This surge reflects massive state investment in pharmaceutical R&D, streamlined regulatory reforms, and an expanding patient pool that enables faster recruitment. These shifts underscore the dynamic nature of global clinical research. To enhance EU's competitive position and streamline regulatory processes, the European Commission's proposed new pharmaceutical package addresses the definition of UMN and the need to support orphan drug development and pediatric formulations. Additionally, the recently implemented new HTA Regulation also addresses unavoidable uncertainties during the pre-approval process (14, 15).

The different regions installed specific fast-track procedures for innovative drugs addressing severe or urgent health conditions. These expedited pathways are designed for drugs that present a significant improvement over existing treatments for serious or life-threatening diseases. In the US, these are identified as Priority Review Designation and Accelerated Approval (both since 1992), Fast Track Designation (since 1997), and Breakthrough Therapy Designation (since 2012) (16). Comparably, the EMA has established various distinct measures to streamline the drug approval process for similar urgent medical products in EU. These include the Conditional Marketing Authorization, the Accelerated Assessment and the Authorization under Exceptional Circumstances (MA for products with less comprehensive data due to the nature of the condition treated, where immediate drug availability is considered beneficial) (all three installed since 2006) (2, 17). Further, based on the Adaptive Pathway pilot initiative (March 2014 to August 2016), the Priority Medicines scheme (PRIME) (since 2016) was set up, which mirrors the Breakthrough Therapy application at FDA (9). In JP, the PMDA offers the Sakigake Designation System (since 2015) and Priority Review (since 1987), both designed to accelerate the approval of innovative drugs addressing UMNs, aligning with global efforts to provide timely access to critical therapies (18, 19). In CH, the NMPA introduced priority review and conditional approval pathways in 2017, formalized in the revised Drug Administration Law (2019), enabling faster evaluation of drugs for serious conditions with no effective treatment (4).

Entering the market as early as possible is crucial for pharmaceutical drug developers. The faster a CT can be set up, the closer they come to a potential MA. Hence, navigating the global regulatory landscape identifying the optimal (adaptive) trial pathway is crucial. The set-up of a fast-track pathway in a certain region not only increases the chances to secure a market position, but obviously affects the patient's early access to the new therapy.

One significant example is Keytruda (pembrolizumab), an anti-PD-1 therapy developed by Merck & Co. Keytruda was first approved in the US in September 2014 under the FDA's Breakthrough Therapy Designation and Priority Review, providing rapid access to patients with advanced melanoma (20). The EMA granted Conditional Marketing Authorization in EU in July 2015 (21). In JP, Keytruda was first approved by the PMDA in September 2016 for unresectable melanoma, and later in December 2016 for PD-L1+ advanced non-small cell lung cancer, under the Priority Review system (22). In CH, Keytruda was approved in 2018 for melanoma, illustrating the country's increasing—but still delayed—integration into global access timelines (19).

However, not all transformative treatments have benefited from such accelerated routes. Despite its groundbreaking nature, Luxturna (voretigene neparvovec), a gene therapy for a rare retinal disease developed by Spark Therapeutics, faced notable regulatory delays across regions. The FDA approved Luxturna in December 2017, making it the first gene therapy approved for an inherited disease (20). The EMA followed with centralized approval in November 2018 (21). In JP, Luxturna received manufacturing and marketing authorization in June 2023, following limited local trial data and extensive regulatory assessment, despite having received Sakigake designation earlier. Reimbursement was finalized in August 2023, nearly 5 years after US approval (22). CH has yet to approve Luxturna as of 2025, despite multiple ongoing trials and regulatory interest (23).

This staggered timeline illustrates how regulatory divergence can delay patient access, even for first-in-class therapies. The resulting delays in the EU, CH, and JP compared with the US demonstrate that regional regulatory processes shape not only company revenues but also the timeliness of access for patients with rare and life-threatening conditions. More broadly, the geography and sequencing of clinical trials determine which patient populations benefit earliest from innovative therapies. Yet, trial geography represents only one axis of inequity. Additional delays arise both from the regulatory review process between CT completion and marketing authorization, and from the design and availability of Early and Expanded Access mechanisms.

### 3.2 Disparities between the clinical trial end date and the marketing authorization approval

Upon completion of the CT process, regulatory agencies undertake a meticulous review of CT data, manufacturing protocols, and product labeling. This review process involves a detailed benefit-risk assessment, based on the significance of assessed (surrogate) clinical endpoints, including many unavoidable uncertainties. This process can take up to 2 years, depending on the complexity of the regulatory pathway chosen. For sponsors, shorter timelines for regulatory MA are critical, influencing their decision to submit a New Drug Application (NDA) or Biologics License Application (BLA) in specific regions first (24).

In the EU, the regulatory system offers several pathways, depending on the company's scope of marketing strategy; whether centralized through the EMA, decentralized to multiple Member States, or mutual recognition from one Member State to others. The centralized procedure allows a single application to the EMA, leading to a single decision by the European Commission, which is then valid across all Member States. This pathway is particularly beneficial for innovative therapies. The Committee for Medicinal Products for Human Use (CHMP) provides recommendations that can support compassionate use programs (CUPs) or open-label extensions, helping Member States to adapt treatments while final regulatory approvals are still pending. Although CHMP guidance complements national laws, the discretion to implement such programs rests with individual Member States. The EMA's centralized procedure typically takes up to 210 days of active review time, though clock-stops for further information requests can extend the total timeframe. An accelerated procedure reduces this to 150 days for drugs of major therapeutic interest (25).

In the US, the FDA uses a centralized system where sponsors submit a NDA or BLA, and the agency assesses the drug's safety and efficacy. FDA's accelerated pathways to facilitate faster patient access to critical treatments expedite the review process significantly, though they still require comprehensive evaluation of CT outcomes. Under the Prescription Drug User Fee Act (PDUFA), the FDA aims for 10 months to complete a standard review, and 6 months for a Priority Review, which is designated for treatments that offer significant advancements or address an UMN (16). This remains longer than the EMA's Accelerated Assessment (150 days). However, the FDA achieves faster market access through a combination of mechanisms, including rolling submissions, real-time reviews, and other expedited pathways that allow parallel regulatory interactions with sponsors (16).

In JP, the PMDA works in close collaboration with the Ministry of Health, Labour, and Welfare (MHLW). JP's review timeline varies but typically aims to conclude within 12 months for standard drugs, while priority drugs undergo an expedited process (3). Programs like the Sakigake Designation are designed to accelerate review timelines further, targeting a 6-month approval period (26). However, in practice, the median approval time remains approximately 332 days (~11 months), making JP's system one of the fastest among major regulatory authorities compared to 333 days at the FDA and 453 days at the EMA, despite the EMA's nominal accelerated procedure (150-day scientific review) (3). A distinctive feature of JP's process is its reliance on Key Opinion Leaders (KOLs) and medical societies, which play a crucial role in advocating for the clinical necessity of novel drugs (3). Additionally, the PMDA may accept overseas clinical data, provided it complies with Good Clinical Practice (GCP) standards. However, this often necessitates additional data or bridging studies to confirm efficacy and safety in the Japanese population, potentially extending approval timelines. While this can delay access compared to other regions, priority review mechanisms remain available for critical therapies (3).

In CH, the NMPA has introduced priority review and conditional approval pathways since 2017, institutionalized in the revised Drug Administration Law (2019). These reforms have significantly shortened review timelines: median approval time for innovative drugs decreased from over 2.5 years before 2015 to approximately 12–16 months by 2021 (5, 6). In addition, mechanisms such as acceptance of foreign clinical trial data and rolling submissions were established, aligning China more closely with FDA and EMA practices while reducing duplicative requirements. These reforms have allowed China to approve multiple first-in-class drugs faster than before, though residual delays remain compared with the US (6).

### 3.3 Disparities between early access programs as an alternative for non-eligible patients

Participation in CTs is often limited by strict eligibility criteria, only allowing a subset of patients to be enrolled based on factors such as disease stage, prior treatments, and overall health status (11). For those unable to participate in a CT, two alternative pathways exist: EAPs and EA. EAPs provide treatment access before regulatory approval, while EA can take place both before and after regulatory approval but before routine clinical use. Despite the variety in terminology, the core principle behind these programs remains consistent worldwide: the ethical provision of investigational drugs to patients not eligible for CT participation, to address an UMN in cases where no other treatment options are available (11).

As outlined in Table 2, the US, EU, CH and JP have established distinct programs to facilitate faster access to investigational drugs as a last resort for patients facing serious or life-threatening conditions. However, disparities exist within and between these regions. Access to these programs depends on the establishment of country-specific pathways, on the willingness of physicians to initiate the application process, and the pharmaceutical companies' readiness to supply the investigational drugs. These variables can significantly impact a patient's ability to receive potentially life-saving treatments.

**Table 2:** Overview of Early Access Programs across major regulatory regions (US, EU, Japan, and China), including their legal basis, eligibility criteria, scope, and emergency flexibility.

**Region**	**Program(s)/legal basis**	**Eligibility**	**Scope**	**Emergency flexibility**
US (FDA)	Expanded Access (IND Subpart I); FDA Form 3926 (single-patient, incl. emergency) (1)	Serious or life-threatening disease; no satisfactory alternatives; benefit–risk supports use	Individual, intermediate-size groups, treatment protocols	Yes—single-patient emergency use; sponsor must submit within 15 business days; cost recovery limited to direct costs (21 CFR 312.8, 2024 guidance)
EU (EMA + MS)	Compassionate Use Programs (CUP, Art. 83 Reg. 726/2004); Named Patient Programs (NPP, Art. 5 Dir. 2001/83/EC)	Serious or chronic life-threatening disease; absence of alternatives; medicine under EMA review/development	CUP = cohorts; NPP = individual patients (national level)	Variable across MS; no harmonized EU emergency use
JP (PMDA/MHLW)	Expanded Access Clinical Trials (EACTs), under Clinical Trials Act (2016)	Patients with serious disease not eligible for ongoing trial; must be embedded in EACT protocol	Cohort-based only; no individual or n-of-1 route	No emergency or single-patient pathway outside EACT protocol
CH (NMPA + Hainan)	National: Conditional Approval/Priority Review (4); Regional: Hainan Boao Lecheng Pilot Zone (2019–)	Severe or rare diseases without effective therapies; Boao enables access to unapproved foreign drugs in urgent clinical need	National = cohorts; Boao = individual patient access permitted	Yes (Boao): accelerated local procedures, >400 imported drugs accessed, with real-world data collection

In the US, the FDA's Expanded Access, also referred to as Compassionate Use (CU), provides a flexible and streamlined approach for patients to gain access to investigational therapies outside of CTs (16). The program offers several pathways, including individual patient access, intermediate-size patient populations' access, and emergency use (16). Between 2010 and 2015, the FDA approved 99% of single-patient EA requests, underscoring the agency's commitment to patient access. The US system, designed to provide quick access to investigational drugs, is one of the most responsive frameworks globally for CU (16).

In contrast, EU presents a more fragmented landscape for early access. Compassionate Use Programs (CUPs) and Named Patients Programs (NPPs) vary significantly across EU Member States (MS) (27). Countries, such as Germany and France, have well-established CUPs that provide broad access to investigational drugs. However, disparities emerge between Northern, Southern, Eastern, and Western Europe, with countries in Southern and Eastern Europe often facing more bureaucratic hurdles, limited resources, and inconsistent implementation of EU guidelines (27).

JP's approach to CU is notably more restrictive compared to the US and EU. The introduction of Expanded Access Clinical Trials (EACTs) in 2016 provided a mechanism for patients with serious or life-threatening conditions to access unapproved drugs (3). However, JP lacks the flexibility seen in the US and EU systems, as there is no provision for single-patient or emergency use under CU. This limitation can delay access to critical treatments for severely ill patients who are ineligible for CTs or other forms of early access (3).

China has developed a hybrid system combining national-level reforms and regional pilot initiatives. At the national level, the NMPA established conditional approval and priority use pathways (2017–2019) that allow patients with severe or rare diseases to access unapproved drugs under controlled conditions (4–6). In parallel, the Hainan Boao Lecheng International Medical Tourism Pilot Zone has since 2019 enabled access to over 450 overseas innovative drugs not yet approved in China, benefitting more than 28,000 patients by 2023 (13, 28). Unlike the US, which has widespread single-patient EA, or the EU, where CUPs are nationally fragmented, China's model blends centralized conditional approval with region-specific pilots, creating a unique but uneven access landscape.

### 3.4 Health system structures and their impact on early access

The architecture of health financing systems substantially influences the functioning and equity of early access pathways. Beyond regulatory frameworks, the design of healthcare systems determines the feasibility and durability of granting early access, particularly in settings where reimbursement is centralized.

In publicly financed systems such as most EU MS and Japan, early or compassionate access is closely intertwined with reimbursement decisions. Once a therapy is introduced before pricing agreements are finalized, it becomes politically and ethically difficult to withdraw if negotiations fail. This dynamic explains why early access in these contexts tends to be more restrictive and controlled. In the EU, this tension is reflected in the fragmented implementation of CUP and NPP, which are conceived as temporary and tightly circumscribed until a positive HTA and pricing agreement is achieved (7, 8). Recent analyses confirm that delays in access across the EU are driven not only by regulatory timelines but also by multi-layered decision processes and constrained budgets, leading to persistent disparities between Member States (12). In JP, the introduction of EACTs in 2016 created a formalized route for patients ineligible for pivotal trials, but the absence of single-patient or emergency access reflects a structural conservatism designed to minimize fiscal and ethical risks associated with potential withdrawal (3).

By contrast, the US exemplifies how a mixed private–public system permits greater flexibility. Because insurers (private and federal) independently negotiate coverage decisions and patients may in principle pay out of pocket, the FDA can authorize Expanded Access requests (including emergency use) without triggering nationwide reimbursement obligations. This structural difference underpins the responsiveness of the US model: between 2010 and 2015, 99% of single-patient Expanded Access requests were approved, often within 24 h (10). However, this flexibility shifts responsibility to patients and providers, potentially exacerbating inequities in access based on insurance coverage or ability to pay (8).

CH represents a hybrid model. At the national level, inclusion of new drugs into the National Reimbursement Drug List (NRDL) requires protracted negotiations with the National Healthcare Security Administration, creating delays similar to those observed in the EU and JP (29). In parallel, however, regional initiatives such as the Hainan Boao Lecheng International Medical Tourism Pilot Zone, operational since 2019, have created an alternative pathway. By 2023, more than 28,000 patients had obtained access to over 450 foreign or unapproved drugs through Boao, demonstrating how localized flexibility can bypass NRDL delays while generating real-world data (RWD) to support subsequent national approvals (13, 28). This dual-track system illustrates how centralized caution and regional experimentation coexist within the same national framework, balancing speed of access with fiscal sustainability.

Taken together, these cases demonstrate that regulatory architecture alone cannot explain disparities in early access. The underlying health system structure (public vs. mixed financing, centralized vs. fragmented reimbursement) critically determines the degree of caution, the flexibility of access pathways, and the political feasibility of withdrawal if evidence or pricing negotiations turn unfavorable. Cross-regional differences in early access are thus as much a function of health system design as of regulatory science.

### 3.5 Navigating the uncertainty of benefit-risk balance in early access pathways

Early access pathways, EAPs and EA mechanisms, represent a critical lifeline for patients with severe or life-threatening conditions, offering access to innovative therapies before they have completed the rigorous regulatory approval process. However, the inherent uncertainty surrounding the benefit-risk profile of these therapies poses a complex dilemma. On one hand, these programs embody the urgency of addressing an UMN; on the other, they introduce significant risks, both for patients and healthcare systems. A pivotal question is: how do we effectively balance this urgency with the precautionary principle of GCP?

This tension often forces regulatory bodies to make high-stake decisions based on incomplete data, particularly in areas like oncology or rare diseases where the demand for solutions is acute. Yet, what is often under-discussed is the need for more adaptive frameworks, regulatory systems that are not only reactive but are proactively designed to evolve with emerging data (7). Current early access frameworks prioritize expedited access, balancing unavoidable uncertainty (including on progression-free and overall survival) by integrating real-time safety monitoring and adaptive benefit-risk recalibration (7). This would allow regulatory agencies to transition from static decision-making to dynamic oversight, where the approval is not a final, irreversible endpoint but part of a continuum that adapts as new patient data emerge.

In the US, the Accelerated Approval program allows early access on the basis of surrogate endpoints, explicitly accepting a high degree of evidentiary uncertainty. However, compliance with post-marketing obligations is uneven, with only about 62% of confirmatory trials completed within agreed timelines, raising questions about accountability in managing uncertainty (12). In Japan, by contrast, the PMDA tends to mitigate uncertainty by requiring additional local data or bridging studies and by relying heavily on the input of Key Opinion Leaders and medical societies. This cautious approach increases evidentiary robustness but often prolongs patient access timelines (3).

The recently (Jan. 2025) implemented Regulation (EU) 2021/2282 on health technology assessment (HTAR) includes Joint Scientific Consultation (JSC) and Joint Clinical Assessment (JCA) to address inherent evidence gaps during the pre-approval process (14). EU Member States' HTA agencies can collaborate with the EMA to mitigate the risks by embracing RWD. Before regulatory MA, they become co-generators for clinical evidence. The scoping process at the start of JCA identifies Member States' HTAs need for specific PICO (Patient, Intervention, Comparison, Outcome) information. The JSC resembles the former Early Dialogues, on the demand of health technology developers (HTDs) (14). This new Regulation aims to accelerate the time between regulatory MA and national reimbursement decisions, which is based on cost-effectiveness analysis. By including JCA and JSC early in the pre-approval process, regulatory agencies strive to gain more mature data, more information on quality of life (QoL) data—both disease-specific and generic—and—where possible—final endpoints during clinical studies to inform the decision-making process. However, single-arm trials including severely affected patients are more likely to suffer from bias and an underestimation of the true uncertainty, and hence might lead to unreliable estimates of treatment effect. Nevertheless, the residual uncertainty surrounding early access should be leveraged to advance not only the scientific and technological complexity (manufacturing and clinical use of such products), but moreover, also regulatory science. They serve cross-indication learnings on the need for other methodologies for evidence-generation, and adapted policy implementation, now increasingly trialed in the field of orphan drugs.

In China, the management of uncertainty has become a central focus of recent reforms. The NMPA introduced conditional approval in 2017 for drugs treating severe, life-threatening diseases or rare conditions, provided early clinical data suggest clear benefit. Continued access is explicitly tied to confirmatory post-marketing studies, under stricter timelines than in the US (5). Moreover, RWD generated in pilot zones such as Hainan Boao Lecheng are increasingly incorporated into regulatory reassessments, creating a feedback loop between early access and evidence accumulation (6, 13, 28). This model illustrates China's attempt to balance urgent patient demand with the precautionary principle by embedding adaptive benefit–risk recalibration into the regulatory process, though concerns remain about the consistent enforcement of confirmatory trial obligations.

The overall aim to provide rapid access to new potentially beneficial pharmaceutical products comes with many challenges. Unavoidable uncertainty is a fundamental component of decision-making regarding (early) access to, and (conditional) pricing and reimbursement of innovative treatments (30). However, in return for early access to drugs with uncertainty about the added value, the new HTA Regulation streamlines the advice of the EMA and the HTA discussions. This results in rethinking stakeholder collaboration. Pharmaceutical developers increasingly assume co-responsibility for managing evidentiary uncertainty, commensurate with the expected revenues from early market entry. The concept of “live licenses” or “conditional approvals” should evolve into an industry standard, where therapies are allowed on the market with the explicit understanding that continued access is conditional contingent on the accumulation and reassessment of benefit-/risk data (7). Also, by getting early access to innovative therapies, patients add significantly to the learning laboratory by generating the evidence, which can serve to progress regulatory efficiency on more common disease therapies (7). These tensions underscore the need for structural reforms in how early access frameworks handle uncertainty. Beyond refining existing procedures, a more systemic redesign is required, one that proactively aligns regulatory flexibility with ethical rigor and global consistency.

### 3.6 Managed Entry Agreements (MEAs) as instruments for uncertainty management

Uncertainty about clinical benefit and cost-effectiveness remains an intrinsic feature of early access to innovative therapies, particularly in oncology, rare diseases, and advanced therapies. Managed Entry Agreements (MEAs) have become a central policy instrument to reconcile the urgency of granting early access with the need to protect the sustainability of health systems. These agreements, whether financial-based (e.g., discounts, budget caps) or outcome-based (e.g., payment contingent on patient response), are increasingly embedded in early access frameworks across jurisdictions (31, 32).

In the EU, MEAs are increasingly used for high-cost therapies, including ATMPs. Several peer-reviewed analyses show that outcome-based or performance-based agreements have been negotiated for some ATMPs (e.g. CAR-T therapies, novel gene therapies), and that payers in countries such as Italy, France, Germany and the UK are more frequently linking reimbursement outcomes or rebates to predefined treatment benchmarks (33, 34). The combination of conditional approvals at the EMA level and national-level MEAs reflects a systemic attempt to balance early availability with evidentiary uncertainty (2). However, fragmentation persists: while countries such as Italy and England have institutionalized outcome-based schemes through registries and dedicated funds, others rely predominantly on financial-based discounts, limiting real-world evidence generation (34).

In the US, formal MEAs remain uncommon, reflecting the fragmented and decentralized payer landscape. Instead, payers increasingly explore payment models for high-cost therapies that attempt to link reimbursement to performance, especially for advanced therapies such as CAR-T cell treatments (35). Recent analyses show that access and affordability challenges are substantial, in part because of uncertainty in long-term benefits and high upfront costs. For example, *CAR-T cell therapies: patient access and affordability solutions* examines how value measurement and real-world evidence are still under development in the US (36). Obstacles such as outcome measurement, data governance, and administrative burden are frequently cited as limiting broader implementation of outcome-based payment models (32). While there are suggestions in literature and stakeholder reports that some outcomes-based contracts are being considered, evidence of widespread diffusion across private insurers or state Medicaid programs remains sparse.

JP relies less on confidential MEAs and more on a centralized cost-effectiveness evaluation (CEE) that adjusts prices after listing. Following a pilot (FY2016–2018), CEE was implemented nationally in April 2019; the evaluation informs value-based price adjustments (not coverage), administered by Chuikyo with technical review by C2H (37, 38). The stepwise adjustment bands are anchored around ¥5 million/QALY (with exceptions), and only the premium portion of price is typically adjusted (38). The much-discussed “Opdivo shock” (large 2017–2018 price cuts under Japan's market-expansion repricing) helped spur the policy debate that led to formal CEE (39). In parallel, JP created a regulated Expanded Access Clinical Trials (EACTs) channel in 2016, run under GCP with PMDA notification; empirical analyses show EACT use is limited and mainly oncology-focused, with procedural burdens noted by industry (3, 40). Overall, JP's uncertainty management relies on rules-based price adjustment and protocolized EACTs, rather than outcomes-based MEAs.

CH has progressively integrated managed-entry elements into national reimbursement via NRDL negotiations. Since 2017, annual reimbursement-linked price negotiations have become the main route to list innovative medicines, producing large price cuts and higher use—e.g., the 2017 policy reduced targeted anticancer medicine costs per DDD by ~49% and increased volumes by 143% (41). Peer-reviewed evaluations also show substantial post-negotiation price drops (median treatment-cost reduction ≈60%) and an explicit move toward value-aligned pricing (42).These changes have improved the availability and affordability of anticancer drugs after NRDL negotiations (29). At the regional level, the Hainan Boao Lecheng pilot creates an early-access pathway for foreign-approved products before national approval and links use to RWD generation; China's regulator (NMPA) formally recognizes pilot RWD for regulatory decision-making (28). By the end of 2023, at least 13 products (9 devices, 4 drugs) had obtained national market approval supported by Lecheng RWE (6). Together, these reforms show China combining NRLD with regional RWD pilots, embedding MEA-like risk-management features within a fast-evolving access framework.

Taken together, approaches to MEAs and related mechanisms vary markedly across regions. The EU has institutionalized outcome- and financial-based agreements within national reimbursement systems, particularly for ATMPs. JP relies instead on a centralized cost-effectiveness evaluation framework to adjust prices post-listing. CH combines steep NRDL price negotiations with regional pilots such as Hainan Boao, linking early access to RWD. The US, lacking a centralized HTA authority, has seen only fragmented, payer-specific experiments with outcomes-based contracts, including for CAR-T therapies. Despite their differences, these models converge on a common function: embedding financial and evidentiary safeguards to reconcile rapid access with uncertainty.

### 3.7 Implications for patients and health systems

Early access frameworks ultimately matter for the outcomes they deliver to patients and for the sustainability of health systems. At the patient level, the US illustrates how permissive expanded access can accelerate time-to-treatment but expose patients to higher residual uncertainty, as only about 60% of FDA-mandated confirmatory trials are completed within recommended timelines (43–46). In the EU, slower and more fragmented pre-approval access reflects the tight coupling of early use with HTA and reimbursement processes; although delays frustrate patients, outcome-based MEAs offer a structured way to generate real-world evidence while ensuring financial protection (47). JP, through Expanded Access Clinical Trials, prioritizes safety and clinical governance by embedding early use in protocolized settings, limiting breadth of access but strengthening confidence in benefit–risk (3). China combines national conditional approval with regional pilots such as Hainan Boao Lecheng, where more than 28,000 patients had obtained early access to unapproved or foreign drugs by 2023, demonstrating rapid access in pockets but also highlighting inequities outside pilot zones (6, 29).

At the system level, these approaches embody different philosophies of risk-sharing. The US tolerates greater evidentiary uncertainty, shifting risk downstream to patients and payers; the EU embeds uncertainty within MEAs and collective reimbursement negotiations; JP relies on cost-effectiveness reassessments and expert societies; and China leverages pilot zones for RWD while using NRDL negotiations to impose steep price cuts.

### 3.8 Approaches to improve early access before regulatory approval

Building on the preceding analysis, reforming early access frameworks requires more than incremental adjustments to regulatory timelines. It demands a structural redesign of how therapeutic innovation is governed under conditions of uncertainty, fiscal pressure, and urgent patient need. The disparities documented across jurisdictions (whether driven by trial geography, divergent regulatory pathways, or fragmented health system structures) reflect the absence of a coherent methodology to manage evidentiary risk before marketing authorization. Addressing this gap requires embedding three interdependent principles at the core of reform: regulatory adaptability, financial sustainability, and equity in access.

First, regulatory adaptability implies moving beyond case-by-case exceptions toward generalizable criteria that clarify *when* early access is warranted, *how* it can be ethically implemented, and *under what evidentiary conditions* it may evolve into full approval. Existing initiatives such as EMA's PRIME, FDA's Breakthrough Therapy designation, PMDA's Sakigake program, and NMPA's conditional approval reforms all share this ambition, yet remain siloed within national contexts, limiting cumulative learning and perpetuating global inequities. A structural shift is needed to transform these parallel programs into interoperable pathways.

Second, financial sustainability must be hardwired into early access design. The expansion of adaptive and conditional approvals without credible post-marketing commitments risks undermining both public trust and payer solvency. Here, MEAs are not peripheral instruments but structural enablers, translating evidentiary uncertainty into accountable payment models. Linking early access to enforceable outcome-based contracts, monitored through interoperable registries, can ensure that accelerated availability is matched by equally accelerated evidence verification.

Third, equity in access requires acknowledging that early access is not merely a regulatory or economic construct but also a distributive choice. Patients excluded from clinical trials or living in jurisdictions without robust EAPs remain structurally disadvantaged. Embedding patient involvement in early access governance, both as contributors of RWD and as co-designers of evidence requirements, can mitigate these disparities and enhance legitimacy.

Innovative tools can operationalize these principles. Adaptive and live licensing models redefine marketing authorization as a provisional entry point, continuously reassessed as evidence matures. Regulatory sandboxes, already piloted in the revised EU pharmaceutical legislation, provide safe environments to test disruptive modalities such as AI-based eligibility algorithms, decentralized trial networks, or gene therapies, while allowing regulators to learn in real time. However, without international coordination, these innovations risk entrenching disparities by advantaging only those jurisdictions with the resources to pilot them.

A genuine transformation requires a trans-regional framework for early access. Mutual reliance pilots between EMA, FDA, and PMDA have shown that cross-agency collaboration can shorten development timelines by several months while reducing duplicative submissions. Extending such initiatives to the NMPA is now indispensable, given China's emergence as the largest hub of global clinical trial activity. Embedding interoperability in data requirements, joint scientific advice, and cross-validated post-marketing obligations would create not only faster but also fairer access.

## 4 Concluding remarks

When you're a patient, you want fast access to potentially disease-altering drugs. It is evident that enhancing CT access and expediting the process from trial completion to market availability is imperative. This will ensure that life-saving treatments reach patients who need them most, in a timely manner. Different regions (US, EU, CH, and JP) have implemented several early access pathways to grant eligible patients this highly-needed early access. However, the timing of early access to innovative therapies significantly differs between the US, the different EU Member States, CH and JP, which leads to healthcare inequities for patients suffering from life-debilitating diseases. The different early access pathways, whether they are based on fast-track CT procedures, EAPs or EA, are by definition designed for fast access to innovative therapies that target an UMN. The unavoidable uncertainty accompanying the early access should be used to progress not only the scientific and technological complexity (manufacturing and clinical use of such products), but moreover, also regulatory science. Recent analyses show that while 62% of post-marketing confirmatory trials in the US are completed within the recommended timelines following accelerated approval, this proportion falls to just 34% in the EU, highlighting a disparity in the enforcement and follow-up mechanisms surrounding early access pathways. In CH, confirmatory study requirements linked to conditional approvals are formally stricter than in the US, but enforcement is still uneven, raising concerns about sustained evidence generation. In JP, by contrast, confirmatory evidence requirements are often reinforced through bridging studies and expert consultations, which reduce evidentiary uncertainty but at the cost of slower access (3). These experiences serve as cross-indication learnings on the need for other methodologies for evidence generation, and adapted policy implementation, now increasingly trialed in the field of orphan drugs.

The new methodologies are making a great leap forward, and could ultimately help to improve regulatory acceleration of more common therapies. The technical inefficiency of conditionally granting access—based on unavoidable uncertainty—is currently mitigated by innovative payment models to avoid allocative inefficiencies, a.o., performance-based agreements whereby pre-agreed data collection further leads to the continued benefit-risk assessment of the drugs. This requires strategies and infrastructure, including collaboration of specialized treatment centers (e.g., the European Reference Networks). The learning opportunity can be maximized by including the knowledge generation on platform technologies and early access of innovative therapies as part of the Commons. To reduce disparities in early access to innovative treatment for patients worldwide, it is key to balance early access and benefit–risk uncertainty through continuous evidence generation and coordinated international frameworks. For this, all stakeholders -including the FDA, EMA, PMDA, and NMPA, alongside HTA bodies, sponsors, trial centers, and patients- need to collaborate more systematically. Only through such global alignment can early access move from fragmented national initiatives to a coherent trans-regional ecosystem that delivers equitable and timely innovation.
